# Information-Driven Docking for TCR-pMHC Complex Prediction

**DOI:** 10.3389/fimmu.2021.686127

**Published:** 2021-06-09

**Authors:** Thomas Peacock, Benny Chain

**Affiliations:** ^1^ Division of Infection and Immunity, University College London, London, United Kingdom; ^2^ The UCL Centre for Computation, Mathematics and Physics in the Life Sciences and Experimental Biology (CoMPLEX), Department Computer Science, University College London, London, United Kingdom

**Keywords:** T cell receptor, docking, ClusPro, HADDOCK, LightDock, ZDOCK, complementarity determining region loops, computational modelling

## Abstract

T cell receptor (TCR) recognition of peptides presented by major histocompatibility complex (MHC) molecules is a fundamental process in the adaptive immune system. An understanding of this recognition process at the molecular level is crucial for TCR based therapeutics and vaccine design. The broad nature of TCR diversity and cross-reactivity presents a challenge for traditional structural resolution. Computational modelling of TCR-pMHC complexes offers an efficient alternative. This study compares the ability of four general-purpose docking platforms (ClusPro, LightDock, ZDOCK and HADDOCK) to make use of varying levels of binding interface information for accurate TCR-pMHC modelling. Each platform was tested on an expanded benchmark set of 44 TCR-pMHC docking cases. In general, HADDOCK is shown to be the best performer. Docking strategy guidance is provided to obtain the best models for each platform for future research. The TCR-pMHC docking cases used in this study can be downloaded from https://github.com/innate2adaptive/ExpandedBenchmark.

## Introduction

T cell receptors (TCRs) occupy a crucial role in the specific recognition of major histocompatibility complex presented antigenic peptides (pMHCs) at the surface of infected cells as part of the adaptive immune response. A process of imprecise recombination of genes in the thymus produces a TCR repertoire that exhibits extraordinarily broad clonal and structural diversity, composed of on the order of 10^11^ sequences (1). This diversity provides the body with a mechanism of targeting a huge array of different antigens while maintaining a high degree of specificity, and makes TCRs intriguing targets for novel therapeutics.

Clinical trials have proven TCR therapy to be a viable strategy against cancer (2), and there is hope that similar attempts will be fruitful against other diseases, such as tuberculosis (3) and HIV (4). Furthermore, TCRs are of considerable interest in the field of vaccine design (5, 6) and in the study of autoimmune diseases (7–9).

Experimental crystallisation and structural resolution of TCR-pMHC complexes can be an expensive and time-consuming process. Computational methods that accurately model these structures could therefore contribute considerably to the study of TCR-pMHC interaction at the molecular level. A number of computational tools that make use of structural templates have been explored in the context of unbound TCR-pMHC (10, 11) and bound TCR-pMHC modelling (12–14). Where no appropriate template is available, protein-protein docking algorithms provide a well-established method for bound complex prediction.

The computational docking community has flourished over the past few decades, producing a wide range of freely available docking software platforms (15), supported and encouraged by the CAPRI blind docking experiment (16). In general, docking algorithms sample thousands of potential poses of the two (or more) binding partners, searching for models that capture the true structure (native solution) of the complex. The models are ranked using a scoring function designed with the aim of reflecting how close a given model is to the native solution.

Rigid body platforms that forgo the modelling of conformational changes, such as ZDOCK (17) and ClusPro (18), achieve rapid search efficiency by sampling conformations in Fourier space (19). Alternatively, coarse-grain Monte Carlo simulations followed by high-resolution refinement for modelling flexibility, notably employed by the RosettaDock platform (20), have also proven effective algorithms in model prediction. An extension of RosettaDock, TCRFlexDock (21), was built with the specific aim of accurately modelling TCR-pMHC structures. Refinement stages for flexible protein modelling are also employed by other platforms, such as HADDOCK (22). Algorithms such as LightDock (23) have approached the problem using normal mode analysis.

In an attempt to improve model accuracy, a number of algorithms have been designed to incorporate additional knowledge of a given docking problem into the sampling and scoring processes. This includes the specification of residues in the unbound proteins that are likely to form part of the binding interface in the bound complex. TCR binding residues are known to nearly always reside in the complementarity determining region (CDR) loop regions. These loop residues bind directly to residues in the presented peptide and to nearby surface residues of the MHC complex in the plane of the peptide groove (24). The conserved features of the binding interface makes these ‘information-driven’ approaches (25) particularly suited to TCR-pMHC modelling. A recent study has compared the capability of four information-driven docking platforms to model antibody-antigen complexes (26), which exhibit similar interface features to TCR-pMHC complexes - namely, the antibody hypervariable loops. This work examines the same four platforms - ClusPro, HADDOCK, LightDock and ZDOCK - as applied to TCR-pMHC modelling.

In order to assess docking software accuracy, experimentally determined structures of the binding partners are required in both unbound and bound form. The unbound partners are passed to the docking software to produce candidate models of the bound complex. These models can then be compared to the bound (reference) structure of the binding partners. A successful model will closely resemble the reference structure. A curated set of 20 TCR-pMHC bound structures with separately solved unbound structures was assembled to form a TCR docking benchmark for testing the TCRFlexDock platform (21). This was subsequently expanded to a total of 30 cases in the TCR3d database (27). An additional 14 cases were identified and used as part of this study.

Varying levels of detail about the interface residues were provided to the algorithms in order to assess how each would perform in the context of limited binding information. Here we present a comparison of the performances of each platform, with the aim of assessing the current state of general purpose docking platforms in the context of TCR-pMHC modelling, and of guiding future researchers in their choice of modelling platform for computational TCR-pMHC docking studies.

## Materials and Methods

### Expanding the TCR Benchmark

The TCR benchmark was expanded to 44 cases of bound TCR-pMHC structures with accompanying unbound TCR and unbound pMHC structures. These docking cases were found by comparing all available unbound TCR structures and all bound TCR-pMHC structures from the STCRDab database (28) with all ‘pMHC-like’ structures from the PDB (29) found through a keyword search (‘MHC’ and ‘HLA’). Structures with a resolution worse than 3.5Å were omitted. Sequence alignment was performed between the TCR chains of the bound TCR-pMHC and unbound TCR structures. Similarly, sequence alignment was performed between the peptide and MHC chains of the bound TCR-pMHC and unbound pMHC structures. Results with high sequence similarity were kept to produce a number of candidate cases containing a bound TCR-pMHC structure and likely matching unbound TCR and pMHC structures. These cases were manually validated, resulting in 44 TCR-pMHC docking cases, shown in **Supplementary Table S1**.

As the with original TCR benchmark (21), structures that included other proteins complexed with the identified structures were omitted. The 2IAM and 2IAN cases, which feature in the original benchmark, are the exception - retained despite the superantigen bound to their pMHC structures (1KLG and 1KLU), as the superantigen does not significantly interact with the peptide or TCR binding site. The superantigen was removed from the structure before docking.

Most structures with missing atoms or residues around the binding interface were also omitted from this study. Those that were retained include the pMHC structure of case 2NX5 (1ZSD; 3 missing side chain atoms in one peptide residue), the TCR structure of case 2NX5 (2NW2; six missing atoms of an absent serine residue and 5 missing atoms in two adjacent residues), the pMHC structure of case 2OI9 (3ERY; 6 missing side chain atoms in one peptide residue), and the pMHC structure of case 2PXY (1K2D; 6 missing side chain atoms in one peptide residue).

The Modeller program (30), version 9.25, was used to add the missing atoms and residues to the above structures, using the automodel class. In each case, ten models were produced, and the model with the best Modeller DOPE score was retained as a repaired model.

Each structure was cleaned before docking. This process involved: reducing the structure down to a single TCR-pMHC, TCR or pMHC, in cases where multiple complexes were crystallised together; removal of solvent and other small molecules; and removal of disordered atoms. Chains were relabelled to conform to a convention set by the original TCR benchmark: TCRs with chain IDs ‘D’ and ‘E’; MHC molecules with chain IDs ‘A’ and ‘B’; and peptides with chain ID ‘C’. TCRs were renumbered according to the IMGT numbering scheme to ensure CDR loop regions had consistent residue IDs. Cleaning of structures was performed using the Bio.PDB (31) Biopython (32) module. TCR renumbering was performed using the ANARCI (33) and Bio.PDB Biopython python modules. Additionally, TCRs and pMHCs were randomly translated and rotated using the PyMOL (http://www.pymol.org) api to avoid initial orientation bias. This clean set of structures is available at https://github.com/innate2adaptive/ExpandedBenchmark.

### Benchmark Difficulty Evaluation Criteria

The difficulty of modelling protein-protein interfaces is recognised to be greater for flexible surfaces, which can adopt very different conformations in bound and unbound states. A difficulty classification was calculated for each docking case using the interface root-mean-square deviation (I-RMSD) between bound and unbound contact surfaces and the fraction of non-native contacts in the complex (F_non-nat_) (34, 35). The criteria for each difficulty class is given in **Table 1**. I-RMSD and F_non-nat_ values were calculated using the Bio.PDB (31) Biopython (32) module, and are shown for each complex in **Supplementary Table S1**.

**Table 1:** Docking difficulty is described by the I-RSMD and fraction of non-native contacts.

Difficulty	I-RMSD (Å)	F_non-nat_
Rigid	≤ 1.5	≤ 0.4
Medium	> 1.5 and ≤ 2.2	–
	or	
	≤1.5	> 0.4
Difficult	> 2.2	–

### Docking Scenarios

**Figure 1:**
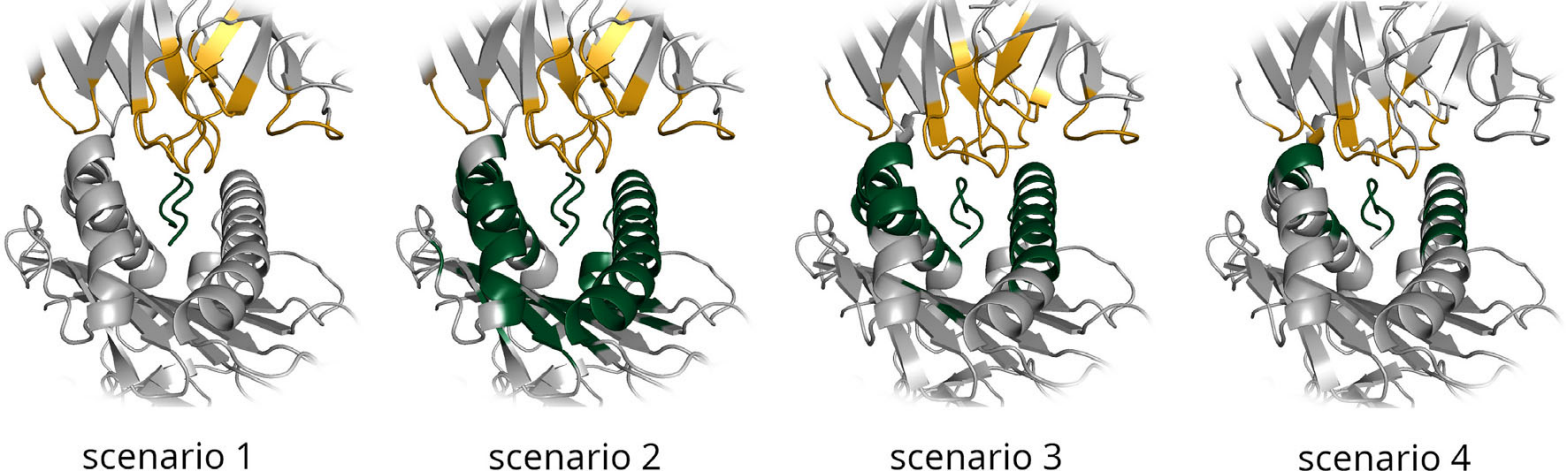
Residues selected as being involved in the binding interface are shown for each of the four docking scenarios for an example TCR-pMHC interface. TCR residues are highlighted in gold and pMHC residues in green.

ClusPro, HADDOCK, LightDock, and ZDOCK all allow additional information about the binding interface to be provided to their algorithms to improve modelling. Four scenarios that provide various levels of information were constructed to assess the difficulty of obtaining high quality bound models with limited knowledge of the true binding interface. The four scenarios are illustrated in **Figure 1** and are described below.

Scenario 1 is the simplest docking scenario. Only the CDR loops and the peptide residues are provided as information about the binding interface. This information is very easy to obtain - all that is required is that the TCR unbound structure is renumbered according to the IMGT numbering scheme, to ensure the CDR loop residues always possess the same range of residue IDs.

Scenario 2 provides a vague definition of the binding interface, composed of the CDR loop residues in the unbound TCR structure, and the peptide and selected surface MHC residues in the unbound pMHC structure. MHC residues were selected from the unbound pMHC structure as any residues within 9Å of the peptide.

Scenario 3 also provides a vague definition of the binding interface, but uses information from the known reference structure. This would generally not be available when performing a TCR computational docking experiment. Any residues in the pMHC component of the bound structure within a distance of 9Å from the TCR binding partner were selected as binding residues, along with the TCR loop residues as before.

Scenario 4 provides the most accurate available information about the binding interface. This ‘real interface’ information includes all TCR residues and pMHC residues that are within a distance of 4.5Å from their corresponding binding partner in the bound reference model.

For each scenario, selected residues were submitted along with the unbound structures to the docking software to produce a set of bound TCR-pMHC models.

It should be emphasised that the binding residue information for Scenarios 1 and 2 is obtained from the unbound TCR and pMHC structures - that is to say that these scenarios represent the case where the structure of the unbound components are known, but the bound reference structure is unknown. In contrast, Scenarios 3 and 4 rely on information from the bound reference structure, which is generally unavailable for a computational docking experiment.

CDR loop renumbering and residue selection based on distances were performed with the ANARCI (33) and Bio.PDB (31) Biopython (32) modules.

### Docking Settings

Models from ClusPro were generated using the ClusPro webserver (https://cluspro.org) (18) with the default settings. Binding residue information was submitted as attractive residues.

Models from ZDOCK were generated using the ZDOCK webserver (http://zdock.umassmed.edu/) (17) version 3.0.2 with the default settings. Residues in both the TCR and pMHC unbound structures that were not involved in the binding for each scenario were supplied to the blocked residues list.

Models from LightDock were generated using LightDock v0.8.0 (36) and run on the UK Materials and Molecular Modelling Hub. Default settings were used for each run - 400 initial swarms, 200 glowworms per swarm and 100 simulation steps. To allow for modelling of flexibility, the Anisotropic Network Model (ANM) mode was activated to calculate the first 10 non-trivial normal modes for both receptor and ligand. The default fastdfire function, a fast C implementation of the DFIRE scoring function (37), was used for model scoring. LightDock allows for the specification of both active and passive restraints. For Scenario 1, TCR loop residues were provided as active and peptide residues as passive; for Scenario 2, TCR loop residues were provided as active and pMHC residues as passive; for Scenario 3, TCR loop residues were provided as active and pMHC residues as passive; for Scenario 4, TCR and pMHC residues were all provided as active.

Models from HADDOCK were generated using the HADDOCK webserver (https://haddock.science.uu.nl) (38) version 2.4. For each scenario, the default sampling settings were used: 1000 models for the rigid-body (it0) stage and 200 models for the flexible (it1) and water refinement stages. While it has been recommended that sampling should be increased when less information about the binding is available, a recent benchmarking of antibody structures using HADDOCK did not show an improvement when sampling was increased compared to the default parameters (26). For Scenarios 1, 2 and 3, the random removal of restraints was set to the HADDOCK default of 50% for each docking run. For Scenario 4, the random removal of restraints was disabled. For each scenario, binding residues were specified as either active or passive residues. For Scenario 1, the CDR loops were specified as active and the peptide as passive. For Scenarios 2 and 3, the CDR loops were specified as active and the pMHC residues as passive. For Scenario 4, residues selected in both the TCR and in the pMHC were specified as active.

For every platform, the unbound TCR was submitted as the receptor structure, and the unbound pMHC was submitted as the ligand structure.

### HADDOCK Clustering Parameters

HADDOCK uses the fraction of common contacts (FCC) as a rapid measure for determining similarity between models in its clustering method (39). The default parameters were used for the HADDOCK clustering analysis (FCC cutoff of 0.6 and a minimum cluster size of 4). The average HADDOCK score of the best 4 models of each cluster was used to produce a ranked list of the clusters (22).

### Model Evaluation Criteria

Every model produced for the four scenarios on each platform was compared to the corresponding reference structure and classified as incorrect, acceptable, medium, or high quality, according to the CAPRI evaluation criteria (40, 41), shown in **Table 2**. The interface root mean square deviation (I-RMSD), the ligand root mean square deviation (L-RMSD) and the fraction of native contacts (F_nat_) were calculated using the Bio.PDB (31) Biopython (32) module, with the TCR set as the receptor and the pMHC set as the ligand.

**Table 2:** Docked models are classified as incorrect, or as of acceptable, medium or high quality in accordance with the CAPRI criteria.

Class	F_nat_	L-RMSD (Å)	I-RMSD (Å)
High	≥ 0.5	≤ 1.0	or ≤ 1.0
Medium	≥ 0.3	≤ 5.0	or ≤ 2.0
Acceptable	≥ 0.1	≤ 10.0	or ≤ 4.0
Incorrect	< 0.1	–	–

## Results

### Expanded TCR Benchmark

The TCR benchmark (21, 27) was expanded to 44 docking cases that were used to assess the TCR-pMHC modelling performance of ClusPro, HADDOCK, LightDock and ZDOCK. A summary of some of the features of these cases is provided in **Table 3**. A complete listing of these cases and their difficulty classifications is provided in **Supplementary Table S1**. The small number of TCR-pMHC Class II complexes in the benchmark set reflects the relatively small number of TCR-pMHC Class II complexes in the PDB overall. This can be attributed to the increased technical difficulty in crystallising Class II structures, with chains often falling apart during the experimental process.

**Table 3:** Summary of biological features of the expanded docking benchmark.

Benchmark Feature	Number
Contains MHC Class I	38
Contains MHC Class II	6
Contains human TCR	39
Contains mouse TCR	5
Max TCR sharing among cases	8
Max pMHC sharing among cases	2
Total unique TCRs	20
Total unique pMHCs	40
Total cases	44

### Docking Performance

**Figure 2:**
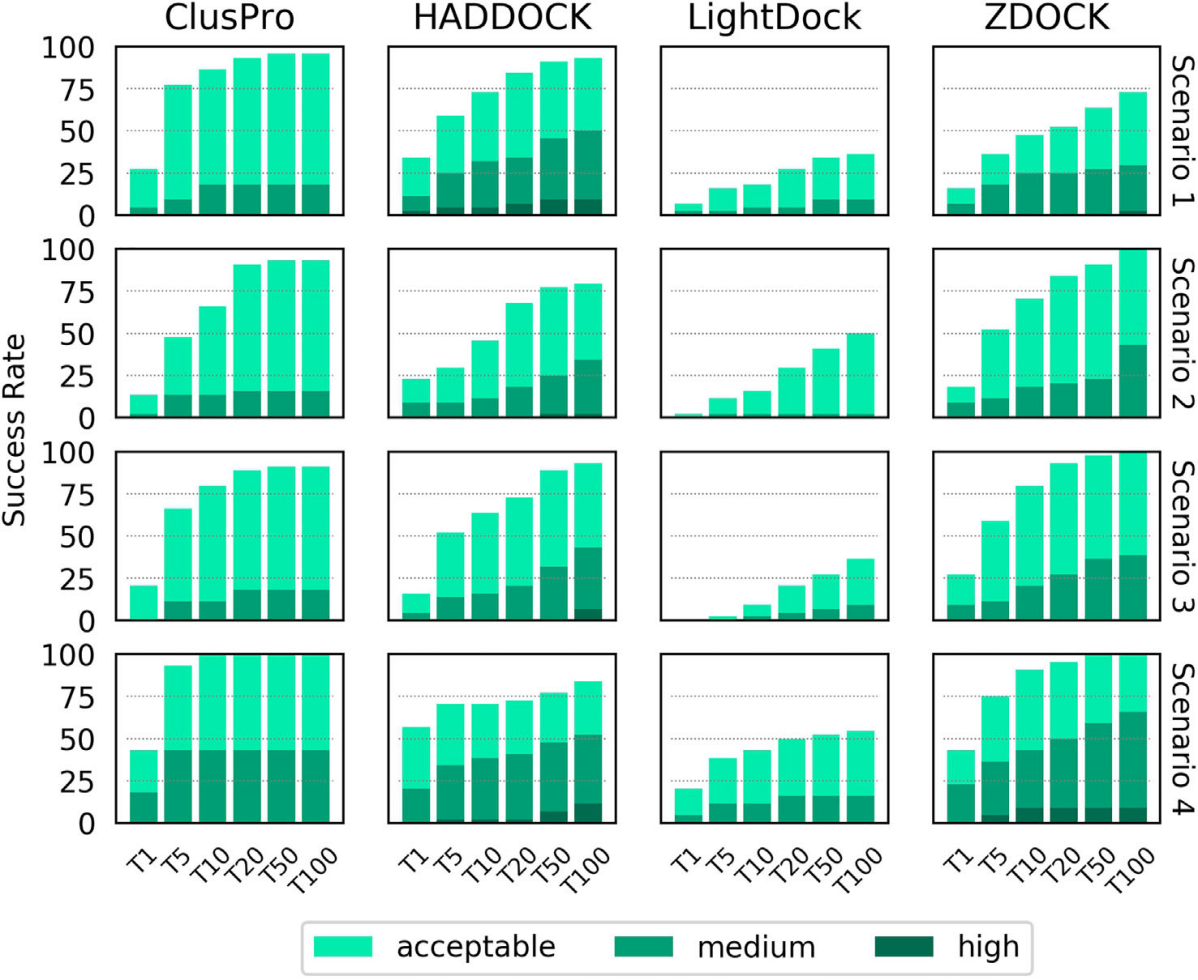
Success rate of the top 1, 5, 10, 20, 50 and 100 ranked models for ClusPro, HADDOCK, LightDock and ZDOCK for each of the four docking scenarios. Colour coding indicates the quality of the best model found in a given set of ranked models according to the CAPRI criteria.

The success rate of each docking platform was calculated as the percentage of cases that featured at least one acceptable, medium or high quality model in the top N ranked models, following an approach used in other benchmarking surveys (21, 26). **Figure 2** shows the success rate for the top 1, 5, 10, 20, 50 and 100 ranked models for each docking tool for each scenario.

When specifying only the CDR loop and peptide residues (Scenario 1), HADDOCK is the best performer for the top ranked model, with a success rate of 34.1%. ClusPro, ZDOCK and LightDock achieved success rates of 27.3%, 15.9% and 6.8%, respectively. When considering the top 10 ranked models, ClusPro is the best performer, with a success rate of 86.4%. HADDOCK, ZDOCK and Lightdock achieved success rates of 72.7%, 47.7% and 18.2%, respectively. When considering the top 100 ranked models, ClusPro is again the best performer, with a success rate of 95.5%. HADDOCK, ZDOCK and Lightdock achieved success rates of 93.2%, 72.7% and 36.4%, respectively. HADDOCK achieved the most medium and high quality models, followed by ZDOCK, ClusPro and LightDock respectively.

The two middle rows of **Figure 2** show the success rate when providing the four docking platforms with the more detailed information of Scenarios 2 and 3, where MHC residues close to the interface are included. The success rate is lower for all top N ranked models for both ClusPro and HADDOCK for both scenarios. The success rate for ZDOCK is improved when using Scenario 2 and Scenario 3 information, and 100% success rate is achieved within the top 100 models. In general, Scenario 3 information (derived from the reference structure) achieves slightly higher success rate than Scenario 2 (derived from the unbound structures).

The final row of **Figure 2** shows the success rate of the four platforms when real interface information is provided (Scenario 4). HADDOCK is the best performer for the top ranked model, with a success rate of 56.8%. ClusPro, ZDOCK and LightDock achieve 43.2%, 43.2% and 20.5% respectively. ClusPro achieves 100% success rate for the top 10 ranked models, and ZDOCK for the top 50 ranked models. Providing the true interface improves performance compared to the other three scenarios for all the top N ranked models for ClusPro, ZDOCK and LightDock, and for the top ranked and top 5 ranked models for HADDOCK.

The LightDock platform performs noticeably worse than the other three platforms for TCR-pMHC modelling. When applying a recommended filtering out of models that do not achieve a minimum number of satisfied restraints, the success rate for Scenarios 1, 2 and 3 decreased, with no acceptable quality models found at all for Scenarios 2 and 3 (**Supplementary Figure S1**). Consequently, the LightDock results shown in **Figure 2** remain unfiltered.

For a given docking case, the ClusPro server generates four sets of models using four different scoring schemes (18), which are compared in **Supplementary Figure S2**. For Scenario 1, the “electrostatic-favored” and “hydrophobic-favored” functions outperform the default “balanced” function (shown in **Figure 2**) for the top ranked model. Additionally, the “electrostatic-favored” function achieves a higher success rate than the “balanced” function for the top ranked models for Scenarios 2 and 4.

**Figure 3:**
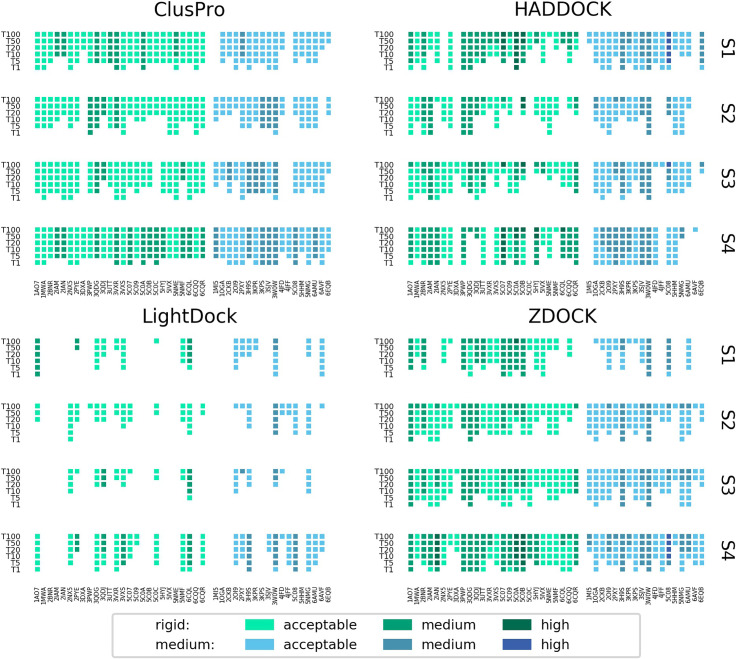
Success rate of the top 1, 5, 10, 20, 50 and 100 models for each complex modelled by ClusPro, HADDOCK, LightDock and ZDOCK for each of the four docking scenarios. Complexes are coloured by their calculated docking difficulty.

**Figure 3** shows the performance of each platform for each scenario per docking case complex, grouped and coloured by difficulty class. ClusPro appears to be the most consistent performer across all docking cases, particularly for real interface information where it predicts at least an acceptable model in the top 5 models for every docking case complex in the study. In scenarios of more limited information. ZDOCK is less consistent across the docking cases, but generates a higher quantity of medium quality models and some high quality models. The performance of HADDOCK exhibits more variation, performing very well for certain cases in Scenario 1 (for example: 5C0A, 3H9S, 3W0W, 5C08), but failing to produce any acceptable quality models in the top 100 for a number of cases in Scenario 4 (for example: 3DXA, 4JFF, 6AMU). For certain cases (for example: 3QDG, 4JFF, 6EQB), HADDOCK performs well given vague information (Scenario 1) but very poorly when given detailed information (Scenario 4). In general, rigid difficulty TCR-pMHC complexes do not seem noticeably easier to predict than medium difficulty complexes.

A visual inspection of models produced by HADDOCK goes some way to explaining the large variation in its success rate, where certain benchmark cases feature an acceptable quality model as the top model, but other cases feature no acceptable model in the top 100 cases. In several cases for Scenario 4, HADDOCK produces what appear to be sensible positions and orientations of the TCR over the pMHC, but with the *α* and *β* chains reversed - that is to say, the TCR *α* chain sits where the *β* chain sits in the reference model, and the TCR *β* chain sits where the α chain sits in the reference model. This is illustrated in **Supplementary Figure S3** for the cases 3DXA, 4JFF and 6EQB. The same pattern was observed for cases 1MI5, 3QDG, 5C0C, 5NMF, 5NMG and 6AVF. HADDOCK attempts to preserve the specified binding residues in its docked models. However, the TCR and pMHC residues it receives are independent - no information is provided about which specific residues are in contact between the binding partners. Therefore, HADDOCK sometimes produces internally high scoring models, with a large number of satisfied restraints, but is blind to the fact that it has positioned the TCR in a reversed orientation.

The docked model results were also grouped and coloured by the MHC class of the complex, shown in **Supplementary Figure S4**. It is difficult to make conclusions about whether any of the platforms are better predictors of TCR-pMHC Class II structure than Class I structure due to the limited number of Class II structures in the benchmark. ClusPro is the most consistent performer across the 6 Class II cases, and shows the best performance for Scenarios 1 and 2, where there is no knowledge of the reference structure. However, no platform shows notably improved performance when modelling Class II rather than Class I, or vice versa.

The success rates of the four platforms were compared with the performance of the TCRFlexDock ‘CDRPep’ protocol (21), which allows for flexibility in the CDR loops and the peptide. The performance of HADDOCK approximately matches the performance of CDRPep for the top ranked model, using Scenario 1 information. When considering the top 10 ranked models, HADDOCK performs worse than CDRPep (73% vs 80%), while ClusPro performs better (86% vs 80%). Using Scenario 4 information, CDRPep is outperformed by ClusPro, HADDOCK and ZDOCK for the top ranked model. When sampling to only the 20 docking cases tested for TCRFlexDock, the success rate for all four general purpose platforms increases (**Supplementary Figure S5**). Across this smaller set of cases, HADDOCK outperforms CDRPep for the top ranked model with Scenario 1 information. ClusPro, HADDOCK and ZDOCK all outperform CDRPep for the top ranked model with Scenario 4 information.

### HADDOCK Cluster Performance

**Figure 4:**
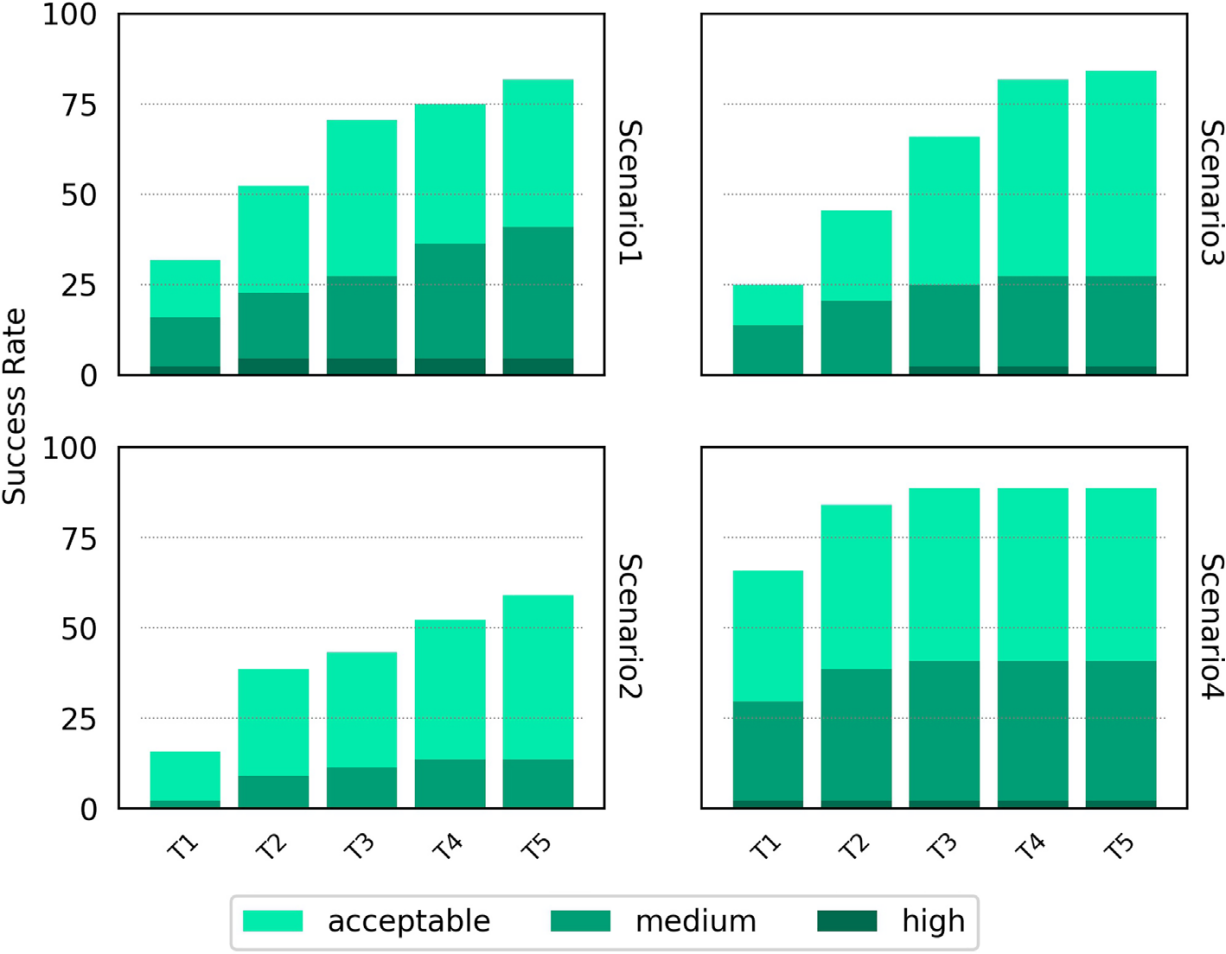
Success rate for the top 1, 2, 3, 4 and 5 ranked clusters for HADDOCK for the four docking scenarios. Colour coding indicates the quality of the best model found in a given set of ranked models according to the CAPRI criteria.

As part of its pipeline, HADDOCK clusters its docked models to provide an alternative set of results to the user - a method that has been shown to improve the success rate of docking algorithms (42). The success rate of the top four members of the top five clusters is shown in **Figure 4** for the four scenarios. A breakdown by individual docking case is shown in **Supplementary Figure S6**. For Scenarios 3 and 4, the success rate for HADDOCK improves using the cluster-based scoring method. There is a decrease in the success rate when providing vague information about the binding from the unbound components, particularly noticeable in Scenario 2. As had been highlighted by previous studies (26), these results suggest that the decision to rely on cluster-based scoring should be carefully chosen based on the detail known about the TCR-pMHC binding region.

### Sampling Performance

**Figure 5:**
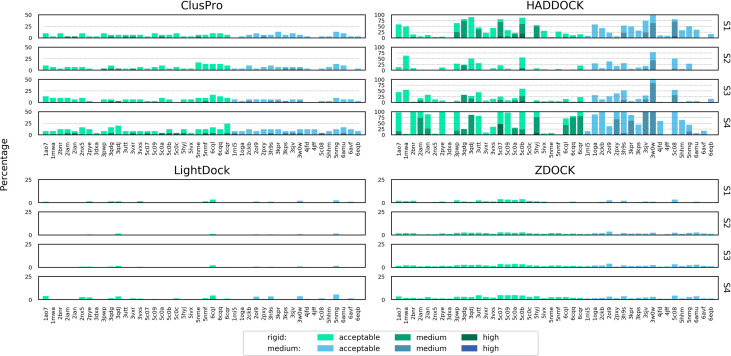
Percentage of total models for each complex of acceptable, medium or high quality according to the CAPRI criteria, shown for ClusPro, HADDOCK, LightDock and ZDOCK for each of the four docking scenarios. Colour coding indicates the quality of models for both rigid and medium difficulty docking cases. The y axis is scaled differently for each docking platform to aid readability.

**Figure 5** shows the number of acceptable, medium and high quality models that were generated as a percentage of the total number of models for a specific docking case for each software and scenario. The sampling performance of HADDOCK in comparison to the other software platforms used is striking. In several cases, when real binding information is provided (Scenario 4), every single model produced is of least acceptable quality (for example: 1A07, 3PWP, 2PXY). The sampling efficiency of HADDOCK in comparison to other software platforms has been reported before and attributed to its use of the binding information to drive the energy minimization and molecular dynamics steps of the simulation, rather than only as part of the model scoring strategy (26). ClusPro is the second best performer, although for no docking case were more than 25% of the models of at least acceptable quality in any scenario.

### CDR3 Loop Modelling

**Figure 6:**
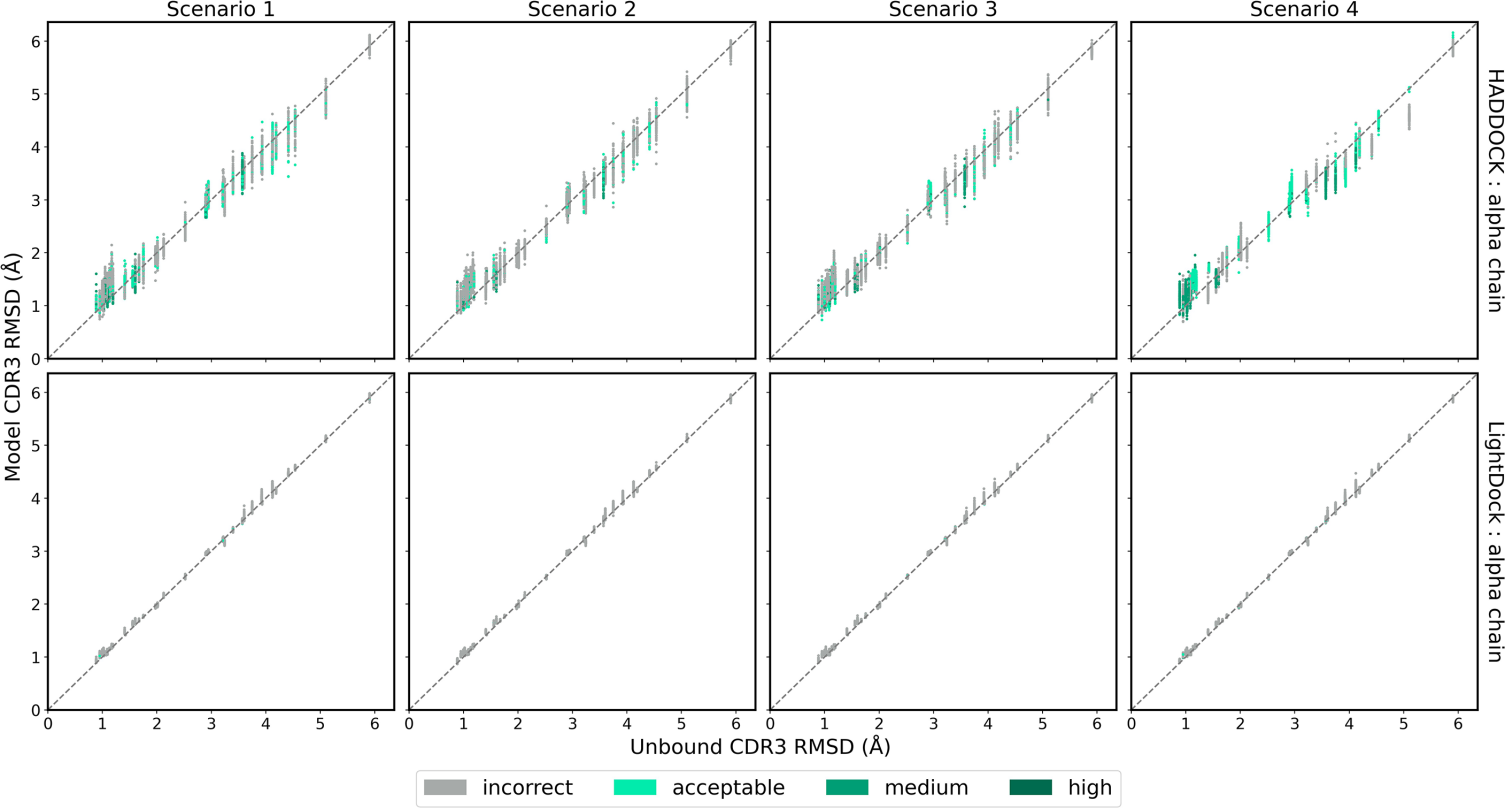
The RMSD of the TCR *α* chain CDR3 loop between the unbound TCR and the reference structure versus that between each of the docked models and the reference structure, for each complex. Loop flexibility modelling by HADDOCK is shown in the top row and by LightDock in the bottom row. Models are coloured by their quality according to the CAPRI criteria.

**Figure 7:**
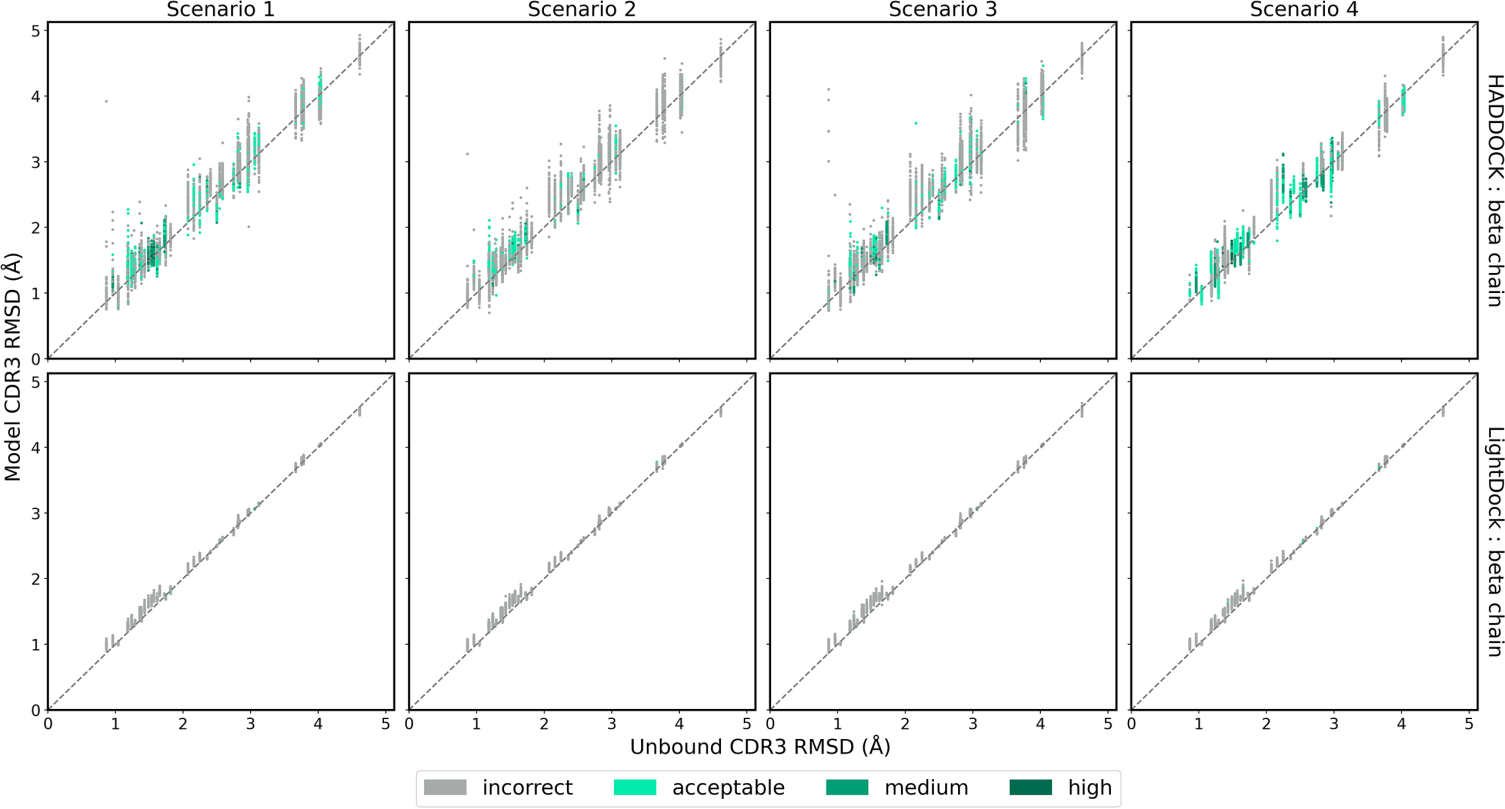
The RMSD of the TCR *β* chain CDR3 loop between the unbound TCR and the reference structure versus that between each of the docked models and the reference structure, for each complex. Loop flexibility modelling by HADDOCK is shown in the top row and by LightDock in the bottom row. Models are coloured by their quality according to the CAPRI criteria.

While ClusPro and ZDOCK are rigid-body platforms, HADDOCK and LightDock allow for conformational change in protein structure during docking. An assessment of the capability of the two software platforms to accurately model TCR CDR3 loops was performed by following a similar strategy recently applied to the modelling of H3 antibody loops (26). The framework residues of the unbound TCR were superimposed upon the bound reference TCR-pMHC structure and the full-atom RMSD of the loop residues was calculated to determine a baseline measure of similarity between the bound and unbound CDR3 loops. The same procedure was carried out for each docked model. The baseline loop RMSD and the model loop RMSD were compared to assess whether the modelled CDR3 loop was closer or further away to the bound structure than the starting unbound structures. **Figures 6** and **7** show the modelled *α* chain and *β* chain loop RMSDs compared to the unbound loop RMSDs for the top 100 models produced for each docking case, respectively. The results are shown for each docking scenario for both HADDOCK and LightDock. Values below the diagonal line correspond to an improvement in the loop conformation, whereas values above the line correspond to a worsening in the loop conformation. Models are coloured according to their quality.

HADDOCK produces models with both improved and worsened CDR3 loop RMSDs. In general, for complexes that undergo low conformation change upon binding, the flexible refinement leads to a worsening of the loop CDR3 RMSD. However, for complexes that undergo greater conformational change, the flexible refinement does not appear to worsen RMSD overall. This pattern is also evident when looking at the refinement as applied to the CDR1 and CDR2 loops, shown in **Supplementary Figures S7–S10**. Loop flexibility in models produced by LightDock are minimal, but generally worsen the RSMD for both *α* and *β* chains.

## Discussion

This study has assessed the ability of four general purpose docking platforms - ClusPro, HADDOCK, LightDock, and ZDOCK - to accurately model TCR-pMHC bound complexes from unbound TCR and pMHC components, mirroring a recently published comparison of the same software suites in the context of antibody-antigen modelling (26). These platforms were chosen as they each facilitate the inclusion of additional information to improve their sets of ranked output models through the specification of binding interface residues. An expanded benchmark set of 44 TCR-pMHC docking cases was identified and used to gauge the performance of each platform.

The results of the four platforms are less impressive when modelling TCR-pMHC structures than when modelling antibody-antigen structures, despite the binding interface interactions being dominated by six flexible loops in both sets of complexes. Antibody-antigen complexes are more frequently modelled than TCR-pMHC structures, and the four platforms have likely been designed, in part, with this in mind. ClusPro, in particular, has a dedicated antibody-antigen modelling feature available as part of its docking suite (43). The relatively low binding affinity between TCR and pMHC is also a phenomenon that is likely to contribute to the poorer performance for TCR-pMHC modelling. The difficulty class of TCR-pMHC docking cases does not seem to have impacted the quality of models produced for the TCR docking benchmark.

All four docking platforms perform best when provided with only the residues known to be directly involved in the interface (Scenario 4). Interestingly, when provided with the vaguest information about the binding interface - simply the residues in the CDR loops and the residues in the peptide (Scenario 1) - ClusPro, HADDOCK, and LightDock all showed a higher modelling success rate than when additionally providing MHC residues likely to be close to the interface (Scenarios 2 and 3). The lower success rate for these scenarios suggests that the specification of a large number of MHC residues may be too heavily restricting the binding mode of the TCR-pMHC models, and that the true binding residues are being lost in a background of residues that are not involved in the binding. In contrast to these findings, ZDOCK improved in accuracy when a broad selection of MHC residues were specified alongside the peptide residues. Rather than scoring models based upon the residues supplied as being involved in the binding, ZDOCK scores models based on the blocking of residues known to not be involved in the binding. This approach is less restrictive in satisfying the MHC residues restraints of Scenarios 2 and 3, and likely explains the improved success rate for ZDOCK for these scenarios compared to Scenario 1. It is therefore important to carefully consider which residues to include when specifying unknown binding information, and how these residues might be used to restrict the binding mode. When specifying binding residues that would be desirable in the output models, a narrow selection is preferable. Alternatively, when specifying residues that should not be blocked by the scoring function, a wider selection may be provided.

ClusPro results improved in accuracy when the weighting of the electrostatic term in the scoring function was increased (“Electrostatic-favored”), and decreased in accuracy when the hydrophobic term was removed (“Van der Waals + electrostatics”). Both hydrophobic and electrostatic interactions have been highlighted as important effects in TCR-pMHC recognition (44), and it would be interesting to explore the effects of these scoring function terms in more detail in future research.

Flexible docking has long been considered an important method for improving modelling accuracy (15). The interaction between the TCR and pMHC is known to be driven by the CDR loops, with the CDR3 loop in particular being key to the recognition process. The modelling of these flexible loops remains a difficult and important problem in the field. TCRFlexDock, a bespoke platform for TCR docking, has shown improvement when allowing for flexibility in the CDR loops and the peptide. However, the two platforms that offer flexible refinement analysed in this study - HADDOCK and LightDock - were unable to consistently improve the conformation of CDR loops regardless of the additional information provided.

Despite an inability to effectively deal with flexibility, the HADDOCK and ClusPro platforms in particular are competitive with TCRFlexDock for TCR-pMHC modelling. HADDOCK achieves approximately the same success rate across the 44 docking test cases using Scenario 1 information as TCRFlexDock achieves across the original 20 benchmark cases for the top ranked model. When limiting the results to only the original 20 benchmark cases, HADDOCK notably outperforms TCRFlexDock using Scenario 1 information. ClusPro outperforms TCRFlexDock when considering the top 10 ranked models using Scenario 1 information for both sample sizes. The higher success rate of ClusPro, HADDOCK and ZDOCK when using Scenario 4 information compared to TCRFlexDock highlights the role accurate binding information can play in TCR-pMHC modelling. Two major advantages of these three platforms are their ease of use and their computational run times. Each platform is supported by a user-friendly online modelling server. The ZDOCK server will often complete a docking case within ten minutes; the HADDOCK server will generally require several hours to complete a docking case. In contrast, TCRFlexDock has been recently reported as taking over 100 hours per complex in its current implementation (14).

Which of the four software platforms is most suitable for the modelling task depends upon the amount of information available and the required quality of the output model. Overall, the HADDOCK platform is the best performer for producing accurate TCR-pMHC complexes as the top ranked model. If the required results are not limited to the top ranked model, ClusPro is the most consistent performer. In the absence of detailed information about the binding interface, it is recommended that users specify binding residues in the form of the CDR loop residues of the TCR and the peptide residues of the pMHC for both HADDOCK and ClusPro. If using ClusPro, users may yield more accurate models by selecting the “Electrostatic-favored” coefficient results. If using ZDOCK, specifying MHC residues close to the interface along with the peptide residues will likely improve modelling results.

Despite some of the successes shown in this study, it is clear that there is opportunity for improvement in the computational docking of TCR-pMHC complexes. In many of the examined docking cases across the four platforms, acceptable (or higher) quality models can be found well outside of the top ranked model. Novel tools for re-ranking or filtering docked TCR-pMHC models could improve the overall success rate. For HADDOCK in particular, provided the researcher is confident in which way round the *α* and *β* chains should sit relative to the pMHC prior to modelling, they might filter out from the results any models where this orientation is not satisfied. Modelling conformational change in the CDR loops and the peptide are very difficult problems, but bespoke approaches would likely produce more accurate models. Finally, it is evident that when exact residues of the binding interface are known, model accuracy dramatically improves. Tools for the accurate prediction of these residues would be an extremely useful aid for information-driven modelling of TCR-pMHC complexes.

## Data Availability Statement

The expanded TCR benchmark docking cases identified and used in this study are available at https://github.com/innate2adaptive/ExpandedBenchmark. The top 200 models generated by each platform for each of the four scenarios and the HADDOCK cluster data have been deposited in the SBGrid data repository (45), and can be downloaded at https://data.sbgrid.org/dataset/825/.

## Author Contributions

TP and BC designed the project and interpreted the data. TP performed the computational analysis. TP wrote the manuscript with contributions from BC. All authors contributed to the article and approved the submitted version.

## Funding

This work was supported by the Engineering and Physical Sciences Research Council (EPSRC) under grant number EP/N509577/1; and by the Royal Free Charity.

## Conflict of Interest

The authors declare that the research was conducted in the absence of any commercial or financial relationships that could be construed as a potential conflict of interest.
