# Supplementary material for: Information-Driven Docking for TCR-pMHC Complex Prediction
Source: Front Immunol. 2021 Jun 9;12:686127. doi: 10.3389/fimmu.2021.686127 (PMC8219952; doi:10.3389/fimmu.2021.686127)
Supplement: Supplementary file 1 [file DataSheet_1.pdf]

Supplementary Material

1 SUPPLEMENTARY TABLES AND FIGURES

Bound Complex	Unbound TCR	Unbound pMHC	MHC Class	I-RMSD	$F_{non\text{-}nat}$	Difficulty
1AO7 *	3QH3	1DUZ	I	1.25	0.33	rigid
1MI5 *	1KGC	1M05	I	1.25	0.48	medium
1MWA *	1TCR	1LEK	I	1.14	0.3	rigid
1OGA *	2VLM	2VLL	I	1.36	0.43	medium
2BNR *	2BNU	1S9W	I	0.72	0.23	rigid
2CKB * ‡	1TCR	1LEG	I	1.17	0.45	medium
2IAM *	2IAL	1KLG	II	0.87	0.24	rigid
2IAN *	2IAL	1KLU	II	0.82	0.3	rigid
2NX5 * †	2NW2	1ZSD	I	1.19	0.37	rigid
2OI9 *	1TCR	3ERY	I	1.1	0.41	medium
2PXY *	2Z35	1K2D	II	1.18	0.55	medium
2PYE *	2PYF	1S9W	I	0.88	0.3	rigid
3DXA * †	3DX9	3DX8	I	1.48	0.39	rigid
3H9S *	3QH3	3H7B	I	1.31	0.42	medium
3KPR *	1KGC	3KPQ	I	1.37	0.55	medium
3KPS *	1KGC	3KPP	I	1.31	0.48	medium
3PWP *	3QH3	3PWL	I	1.24	0.36	rigid
3QDG * † ‡	3QEU	1JF1	I	0.91	0.31	rigid
3QDJ * ‡	3QEU	2GUO	I	0.94	0.28	rigid
3SJV * ‡	3SKN	1M05	I	0.96	0.41	medium
3UTT *	3UTP	3UTQ	I	0.75	0.4	rigid
3VXR	3VXQ	3VXN	I	0.82	0.38	rigid
3VXS * †	3VXQ	3VXP	I	0.89	0.35	rigid
3W0W * † ‡	3VXT	3VXO	I	0.94	0.42	medium
4JFD * †	4JFH	4JFP	I	1.51	0.51	medium
4JFF	4JFH	1JF1	I	1.54	0.52	medium
5C07	3UTP	5C0E	I	0.57	0.15	rigid
5C08	3UTP	5C0F	I	0.65	0.43	medium
5C09	3UTP	5C0G	I	0.59	0.24	rigid
5C0A	3UTP	5N1Y	I	0.5	0.3	rigid
5C0B	3UTP	5C0I	I	0.59	0.25	rigid
5C0C	3UTP	5C0J	I	0.64	0.35	rigid
5HHM	2VLM	5HHN	I	1.42	0.51	medium
5HYJ	3UTP	5C0D	I	0.55	0.34	rigid
5IVX	5IW1	3ECB	I	1.29	0.38	rigid
5NME	5NMD	2V2W	I	1.07	0.34	rigid
5NMF * †	5NMD	5NMH	I	1.05	0.38	rigid
5NMG * † ‡	5NMD	5NMK	I	1.07	0.42	medium
6AMU	3QEU	6AMT	I	1.16	0.41	medium
6AVF * †	6AT6	6AT5	I	1.95	0.72	medium
6CQL * †	6CPH	6CPN	II	0.78	0.23	rigid
6CQQ * †	6CPH	6CPO	II	0.83	0.21	rigid
6CQR * †	6CPH	6CQJ	II	0.85	0.26	rigid
6EQB	4JFH	2GUO	I	1.62	0.55	medium

Table S1. TCR docking benchmark cases of TCR and pMHC structures found in complexed (bound) and uncomplexed (unbound) form, identified in the Structural TCR Database (STCRDab) and the Protein Databank. The I-RMSD, $F_{non\text{-}nat}$ and docking difficulty are provided for each case. It should be noted that in some cases these values differ from those reported in the original TCR Benchmark. This is largely due to differences in which protein chains were selected from the raw PDB data, for cases where multiples of the same protein are present in the crystal structure. In this study, bound and unbound components were chosen such that the RMSD between the two was minimised. Structures can be downloaded from https://github.com/innate2adaptive/ExpandedBenchmark.
* TCR docking cases that feature in the TCR3d database;
† Cases that differ in I-RSMD score to those in the TCR3d database;
‡ Cases that differ in docking difficulty category in the TCR3d database.

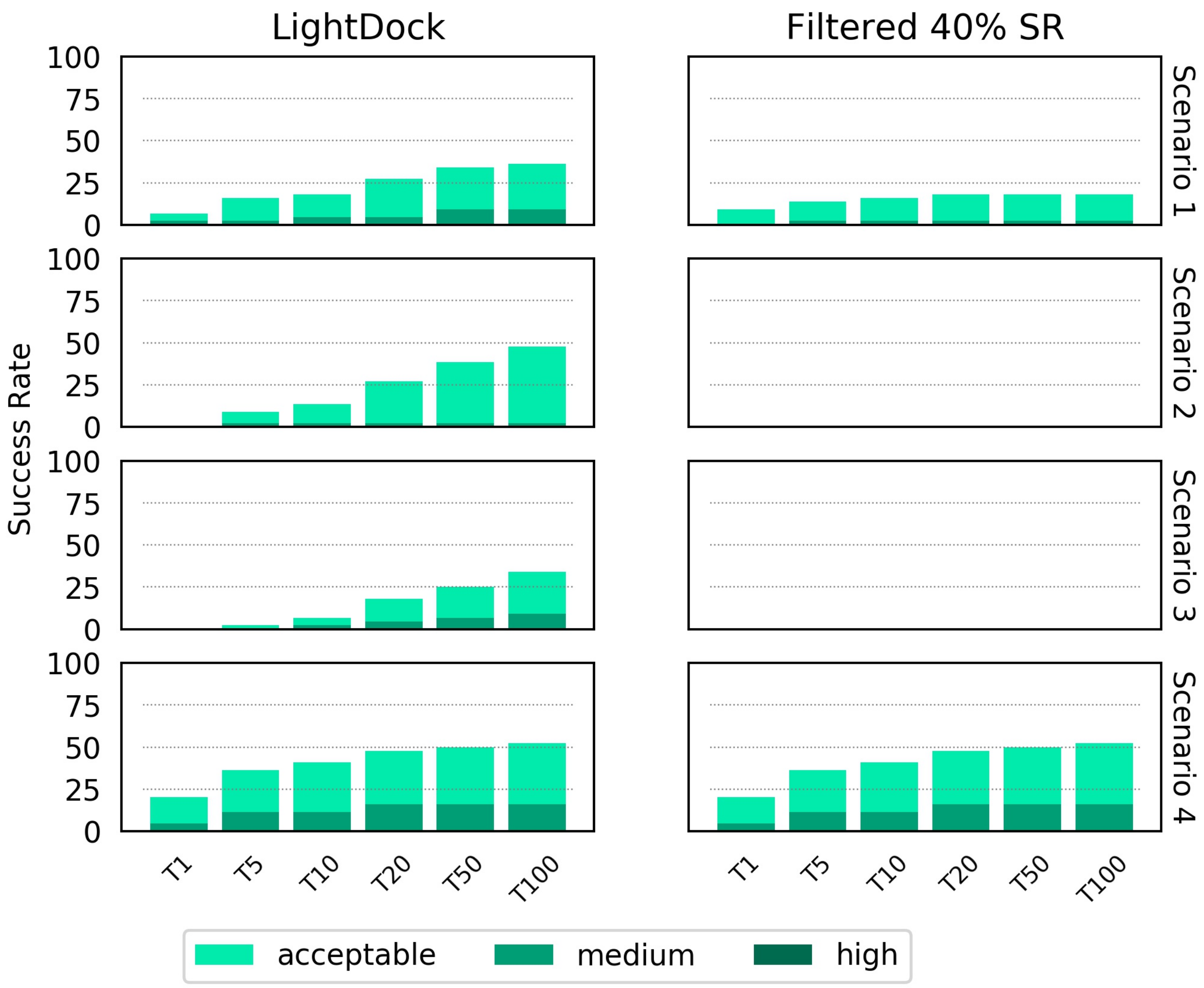

Figure S1. The docking success rate is shown for LightDock without the recommended filtering of models by the satisfaction of spatial restraints as in Figure 2 (left), compared to the same results filtered by the satisfaction of at least 40% of spatial restraints (right). In general, filtering has a negative impact on the TCR docking results, with many models of acceptable or higher quality lost in the filtering, especially for Scenarios 2 and 3.

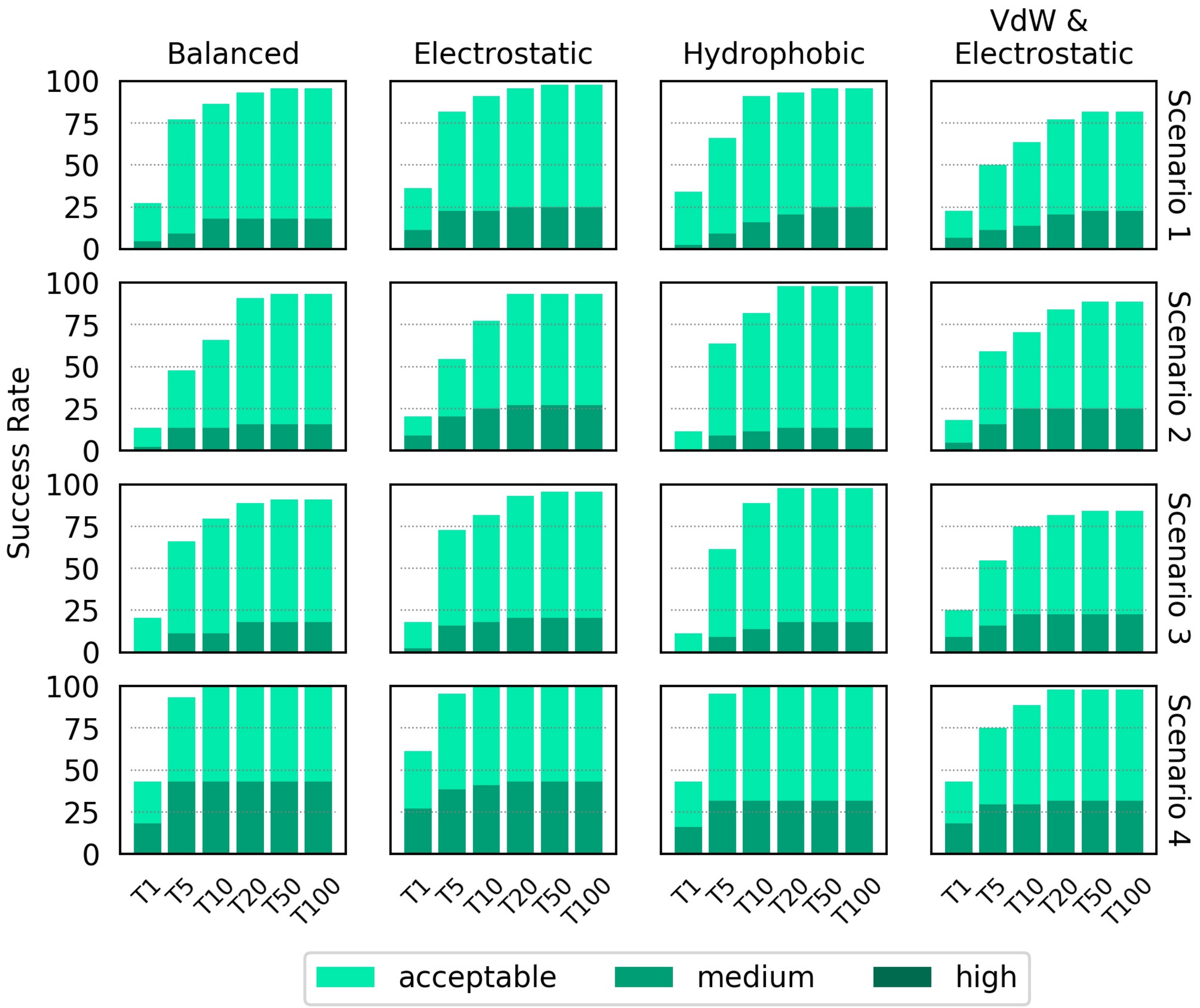

Figure S2. The docking success rate is shown for ClusPro using the four scoring functions provided by the ClusPro webserver. The first column, 'balanced', shows the ClusPro success rate as in Figure 2.

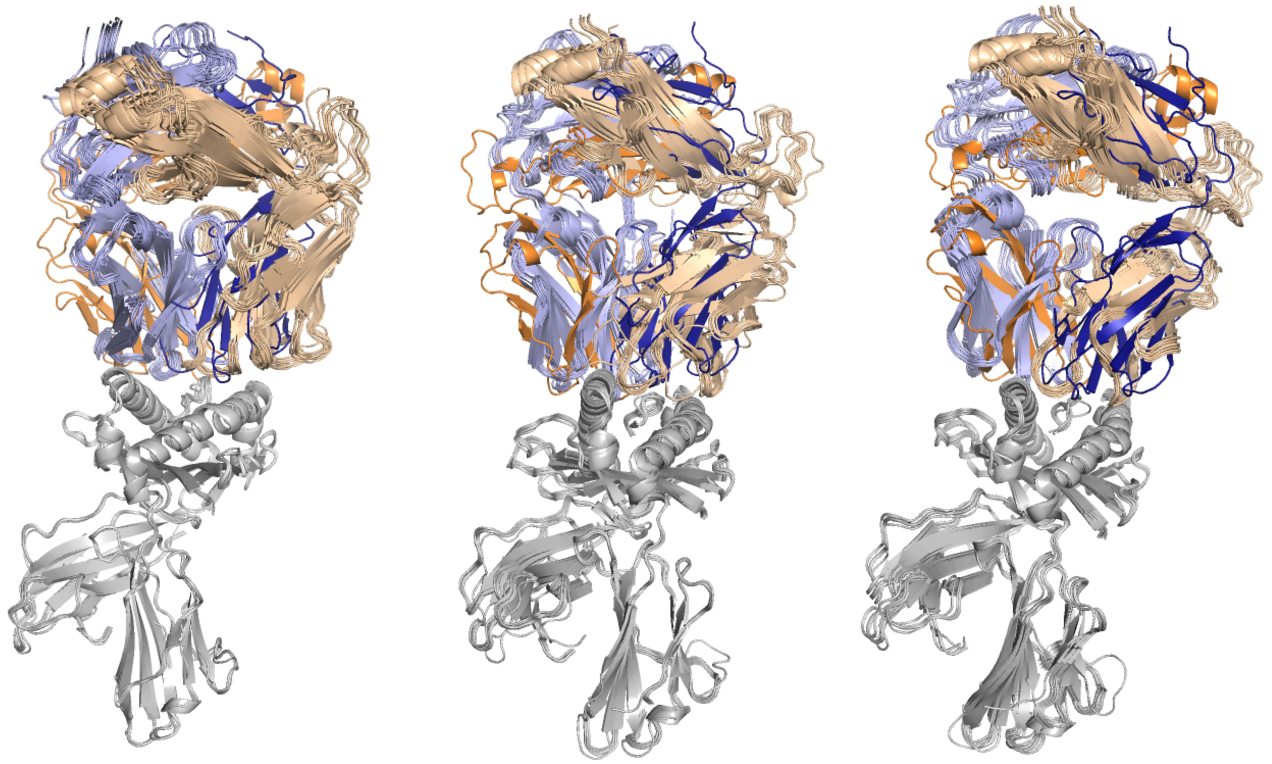

Figure S3. Top 10 models produced by HADDOCK using Scenario 4 information and the reference structure for three docking cases: 3DXA (left), 4JFF (middle) and 6EQB (right). For each case, the pMHC chains in the models are superimposed onto the pMHC chains of the reference structure. TCR chains are coloured light orange and light blue in the docked models, and dark orange and dark blue in the reference structures. For these cases, each of the top 10 predictions by HADDOCK has the TCR reversed in orientation relative to the reference structure. The same pattern can be observed for the cases 1MI5, 3QDG, 5C0C, 5NMF, 5NMG and 6AVF.

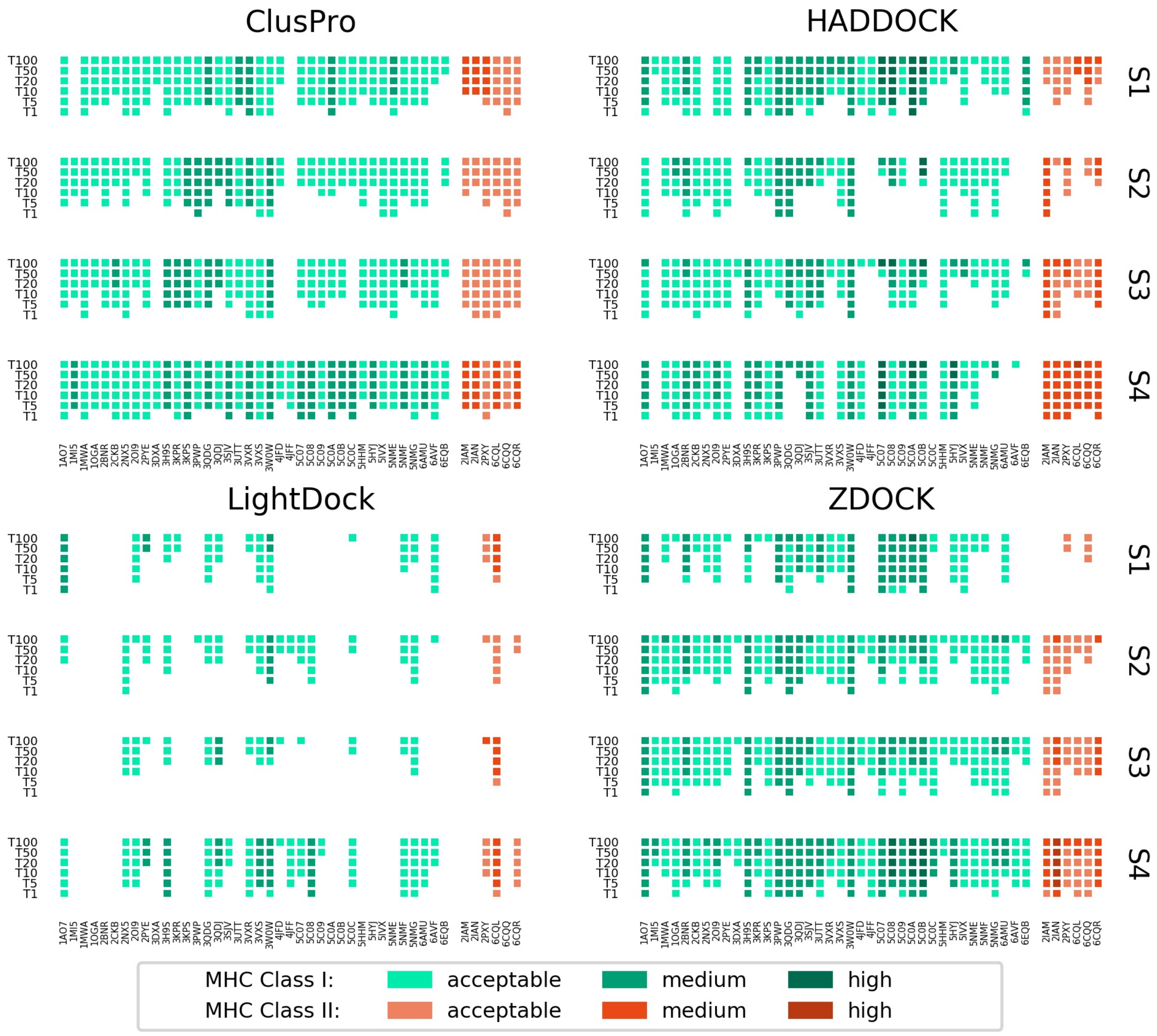

Figure S4. Success rate of the top 1, 5, 10, 20, 50 and 100 models for each complex modelled by ClusPro, HADDOCK, LightDock and ZDOCK for each of the four docking scenarios. Complexes are coloured by their MHC class.

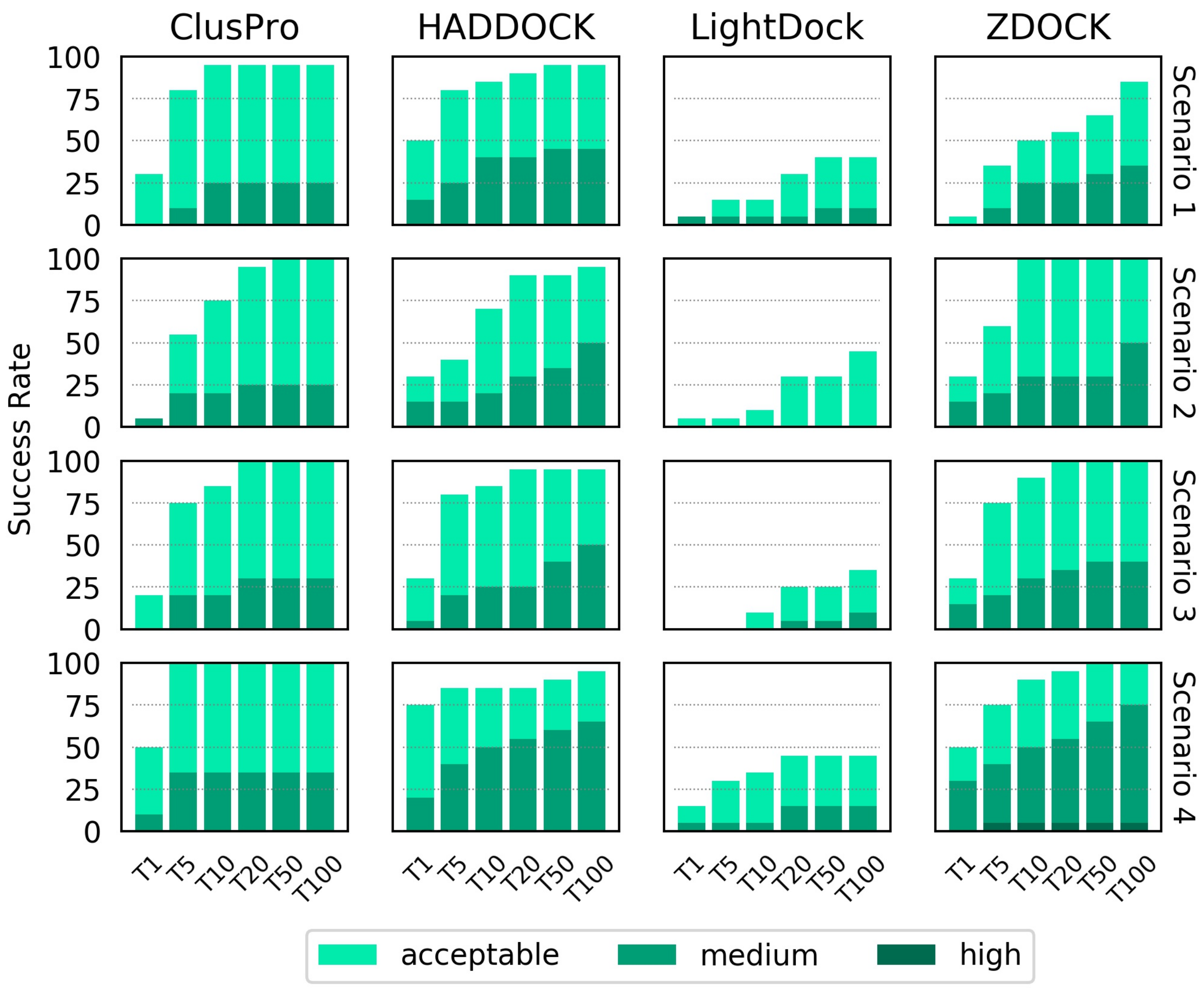

Figure S5. Success rate of the top 1, 5, 10, 20, 50 and 100 ranked models for ClusPro, HADDOCK, LightDock and ZDOCK for each of the four docking scenarios for the 20 docking cases tested against the TCRFlexDock platform. Colour coding indicates the quality of the best model found in a given set of ranked models according to the CAPRI criteria.

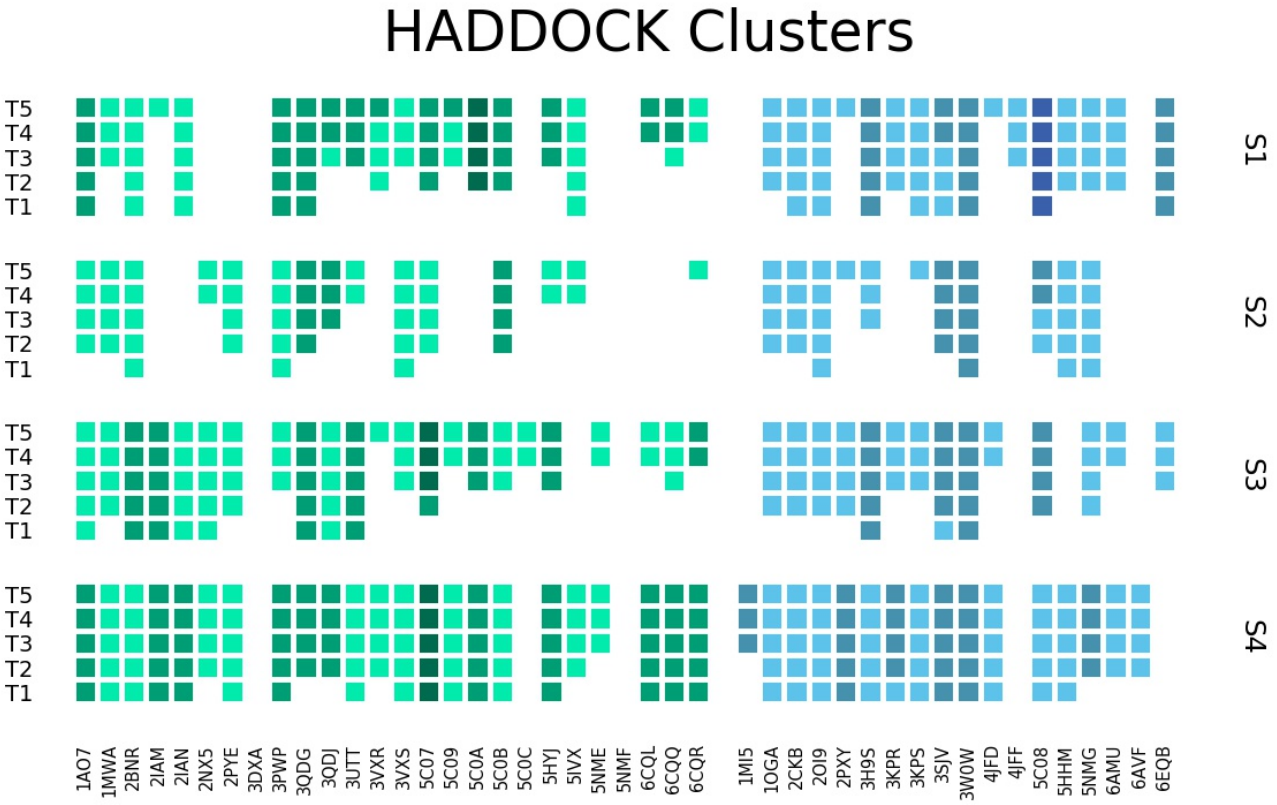

Figure S6. Success rate for the top 1, 2, 3, 4 and 5 ranked clusters for each complex modelled by HADDOCK for the four docking scenarios. Colour coding indicates the quality of the best model found in a given set of ranked models according to the CAPRI criteria.

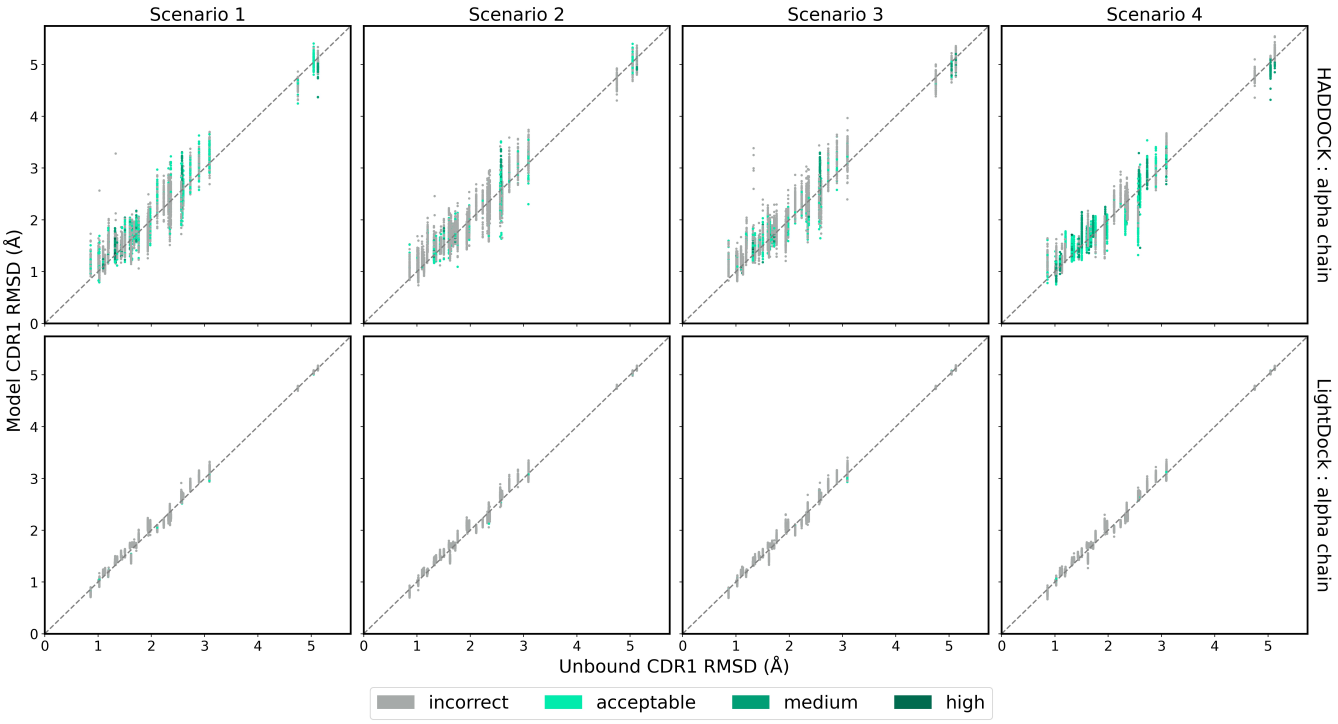

Figure S7. The RMSD of the TCR α chain CDR1 loop between the unbound TCR and the reference structure versus that between each of the docked models and the reference structure, for each complex. Loop flexibility modelling by HADDOCK is shown in the top row and by LightDock in the bottom row. Models are coloured by their quality according to the CAPRI criteria.

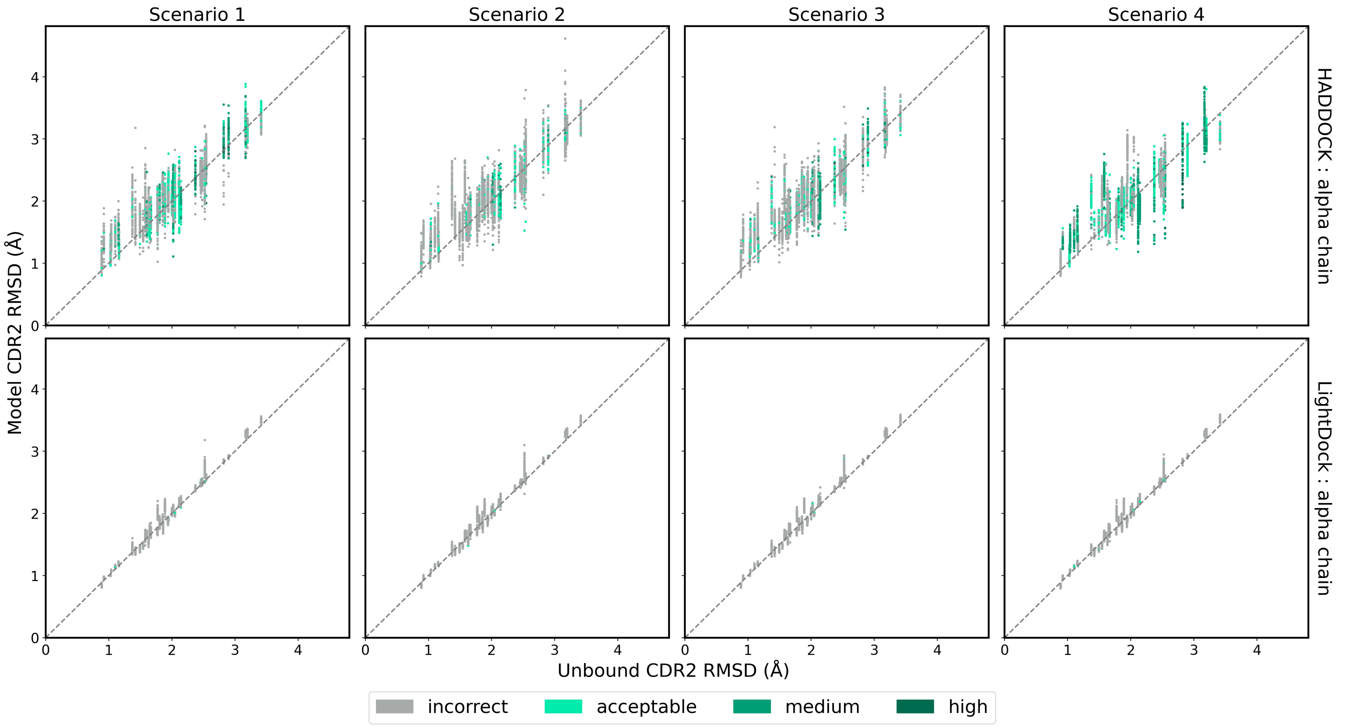

Figure S8. The RMSD of the TCR α chain CDR2 loop between the unbound TCR and the reference structure versus that between each of the docked models and the reference structure, for each complex. Loop flexibility modelling by HADDOCK is shown in the top row and by LightDock in the bottom row. Models are coloured by their quality according to the CAPRI criteria.

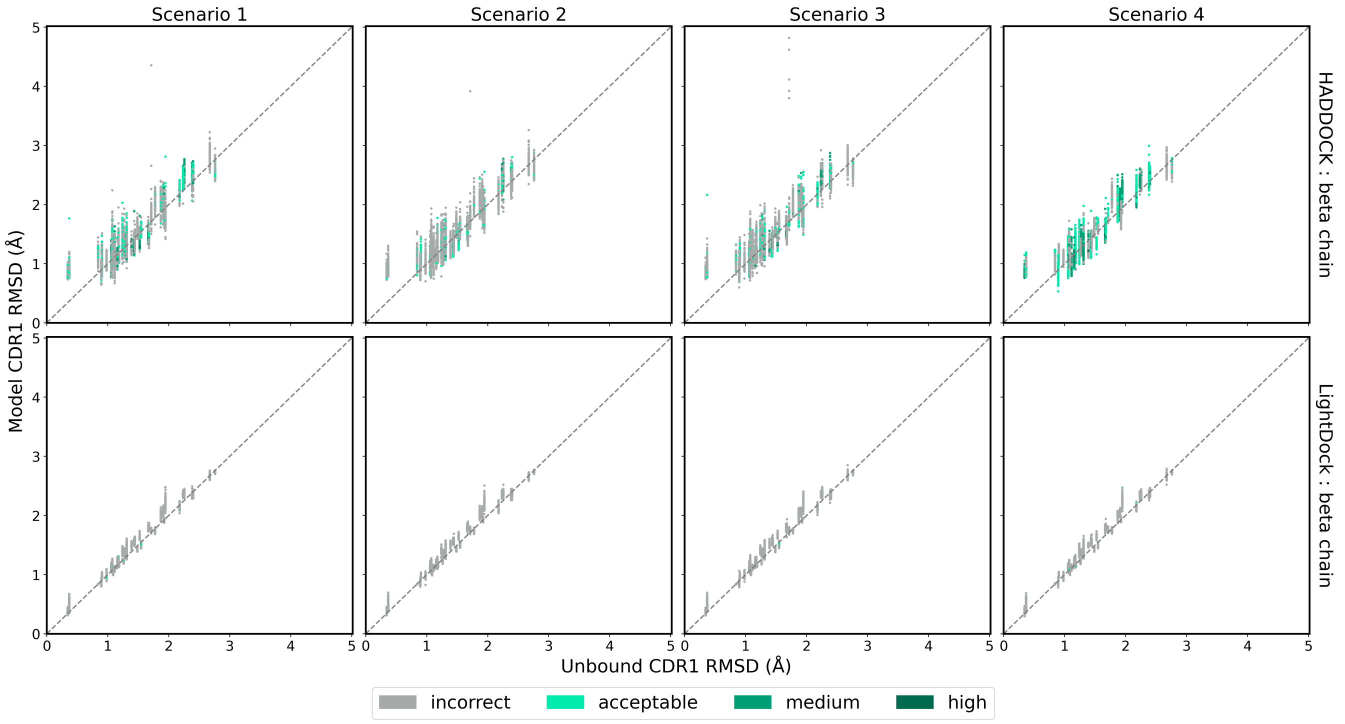

Figure S9. The RMSD of the TCR β chain CDR1 loop between the unbound TCR and the reference structure versus that between each of the docked models and the reference structure, for each complex. Loop flexibility modelling by HADDOCK is shown in the top row and by LightDock in the bottom row. Models are coloured by their quality according to the CAPRI criteria.

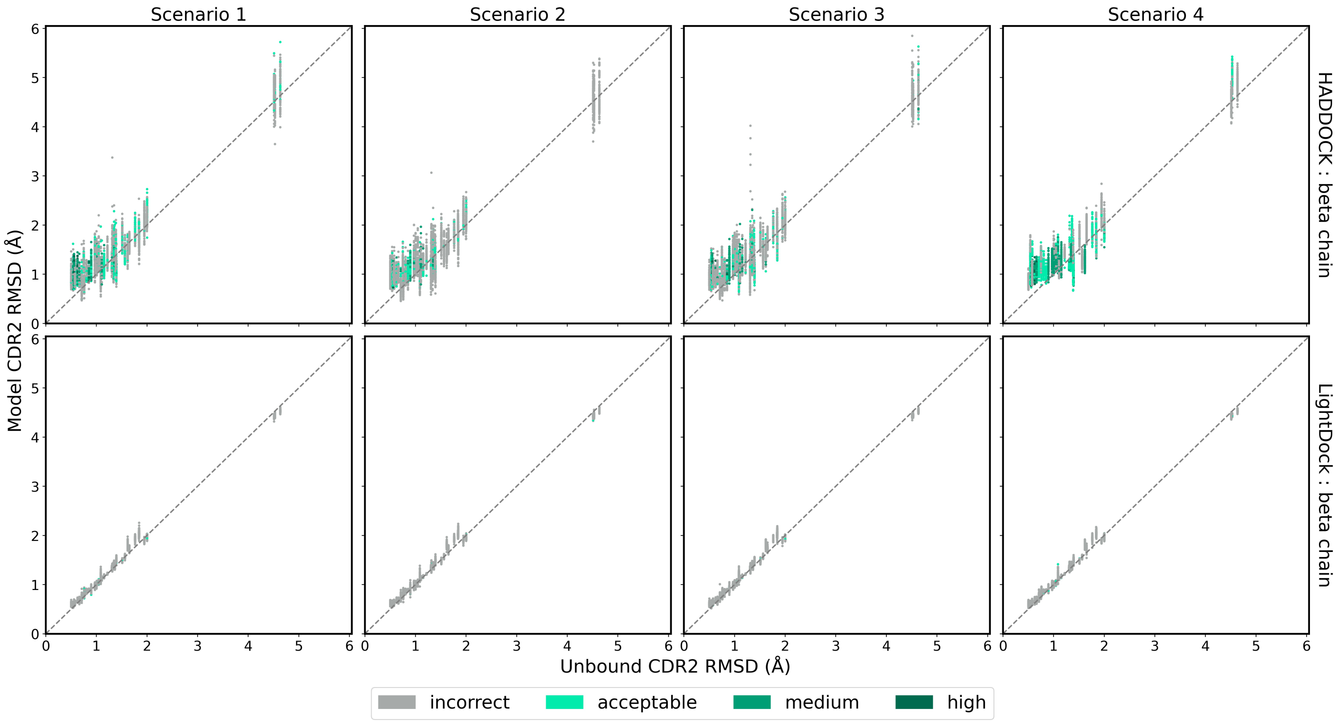

Figure S10. The RMSD of the TCR β chain CDR2 loop between the unbound TCR and the reference structure versus that between each of the docked models and the reference structure, for each complex. Loop flexibility modelling by HADDOCK is shown in the top row and by LightDock in the bottom row. Models are coloured by their quality according to the CAPRI criteria.
